# Study on the size effect of rock burst tendency of red sandstone under uniaxial compression

**DOI:** 10.1038/s41598-024-67464-1

**Published:** 2024-07-16

**Authors:** Feng Chen, Jinyang Du, Jinguo Lv, Chun’an Tang, Yishan Pan

**Affiliations:** 1 School of Mechanics and Engineering, Liaoning Technical University, Fuxin, 123000 Liaoning China; 2 Institute of Rock Instability and Seismicity Research, Dalian University of Technology, Dalian, 116000 Liaoning China; 3 Environmental Engineering College, Liaoning University, Shenyang, 110000 China

**Keywords:** Rock burst, Deformation modulus ratio, Uniaxial compressive strength, Size effect, Energy conversion law, Natural hazards, Solid Earth sciences

## Abstract

The study of rock burst tendency of rock masses with different sizes plays a key role in the prevention of rock burst. Through theoretical analysis, it is proposed that uniaxial compressive strength and deformation modulus ratio are the key mechanical parameters affecting rock burst occurrence. In order to find out the size effect of uniaxial compressive strength and deformation modulus ratio, theoretical analysis and uniaxial compression experiment are carried out on rock samples with different heights, different cross-sectional areas and different volumes. The results show that the smaller the uniaxial compressive strength is, the larger the deformation modulus ratio is, and the more likely rock burst are to occur. On the contrary, rock burst is still not easy to generate. The uniaxial compressive strength of rock samples with different heights, different cross-sectional areas and different volumes increases with the increase of rock sample size. The deformation modulus ratio of rock samples with different heights and different volumes shows an upward trend on the whole, while that of rock samples with different cross-sectional areas shows a downward trend on the whole. The fracture forms of rock are analyzed using the energy conversion law in the process of deformation and failure for three kinds of rock with different shapes and sizes.

## Introduction

Rock burst refers to the dynamic phenomenon of sudden and severe damage caused by the instantaneous release of elastic energy in the rock mass around the working face of coal mine, which is often accompanied by rock mass ejection, loud noise and air wave^1–3^. Rock burst is a worldwide problem encountered in the process of coal mining. As the intensity and depth of coal mining continue to increase, the number of coal mines experiencing rock burst gradually increases^4,5^. The presence or absence of rock burst tendency of rock mass is a prerequisite for rock burst occurrence. Rock burst tendency refers to the ability of the elastic energy stored in rock mass to be released under certain conditions to produce impact failure. Rock burst tendency is a natural attribute of whether rock mass can generate rock burst. The greater the rock burst tendency is, the greater the probability and intensity of rock burst occurrence are^6–10^.

Extensive research exists on the relevant mechanical parameters for evaluating the rock burst tendency of rock mass. These mechanical parameters are mainly divided into four aspects: energy, deformation, failure time and stiffness^9,11^. Among all these mechanical parameters, the most representative specific mechanical parameters are: dynamic failure time, elastic deformation index, bending energy index, stiffness ratio index, effective impact energy index, elastic energy index, brittleness coefficient, etc.^12–17^. Davarpanah et al.^18,19^ believed that the brittleness coefficient of rocks plays an important role in the failure and brittle-toughness transition stress, and pointed out that Hoek brown material constant (mi) has a great influence on the brittleness of rocks.

The mechanical properties of rock mass generally have size effect. Size effect analysis is the foundation for applying laboratory experimental results to rock mass engineering^10,20–22^. Wang^23^, Yang and Xu^24^, Sun et al.^25^, and Zhu et al.^26^ analyzed the size effect of rock long-term strength and the influence of size effect on the strength and deformation under different confining pressures. Zhai^27^ conducted uniaxial compression tests on cubic red sandstone with side lengths of 80 mm, 120 mm, and 160 mm, and pointed out that the probability distribution type of uniaxial compressive strength of rock samples follows the normal distribution. Zhang et al.^28^ obtained the stress–strain curve, strain rate curve and dynamic failure process of rock samples with different sizes using a large diameter split Hopkinson pressure bar system. Zhao et al.^29^ analyzed the waveform spectrum characteristics of acoustic emission signals during single-sided unloading process of granite specimens with different heights. Zhao et al.^30^ analyzed the damage law of sandstone under different height-diameter ratios based on the evolution law of dissipative energy.

The above scholars studied the size effect of stress, strain and acoustic emission of rock mass by different experimental methods. Based on the above research results, a mechanical model of rock burst roadway is established, a point of view is put forward that deformation modulus ratio and uniaxial compressive strength are the key mechanical parameters affecting rock burst occurrence, and the size effect of relevant mechanical parameters for evaluating the rock burst tendency in rock mass are analyzed in detail.

The size effect of red sandstone is very important for engineering design, risk assessment and cost control. The study of its size effect is helpful to accurately select mechanical parameters of rock mass, predict rock burst tendency and reduce the need for in-situ test, thus reducing engineering cost and providing reliable support for engineering design and stability analysis.

## Theoretical analysis of rock burst roadway

### Critical load of rock burst occurrence

**Figure 1:**
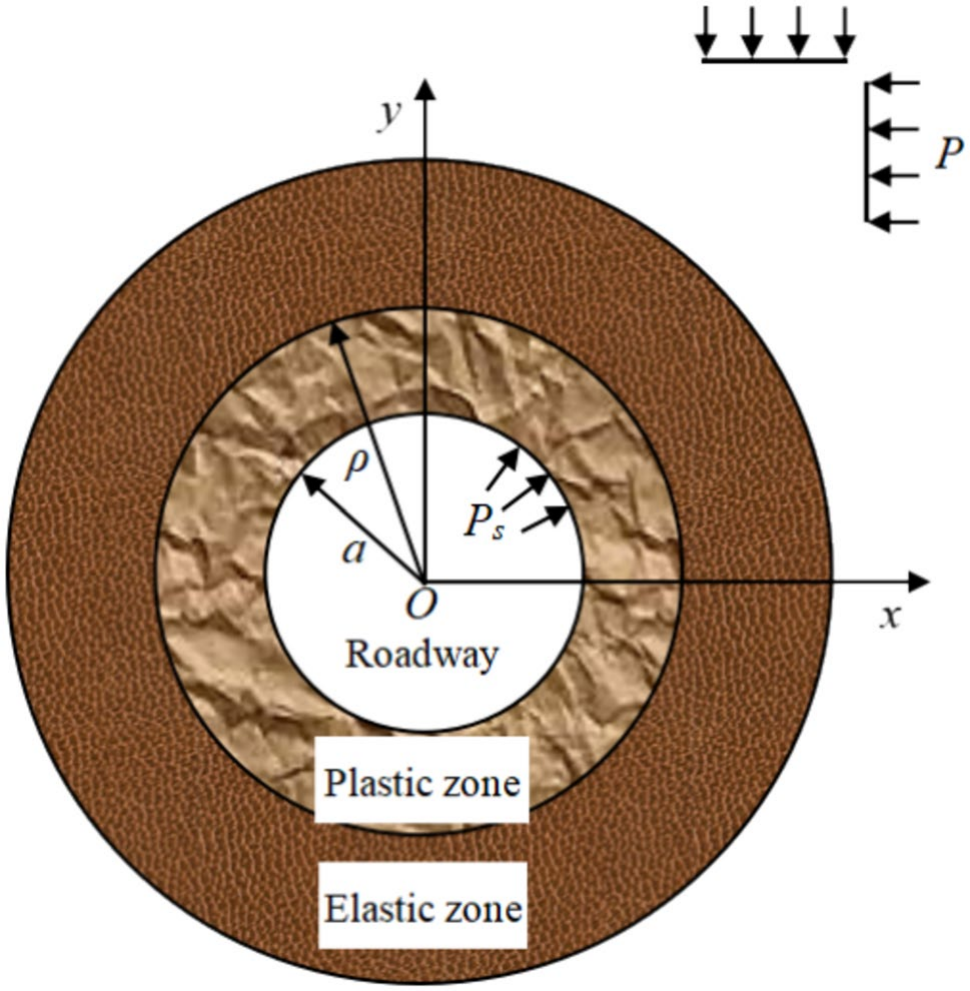
Calculation model of circular cross section roadway.

The shape of the roadway cross section is assumed to be circular with an average radius of *a* in order to facilitate the theoretical derivation, as shown in Fig. 1. The distance from any point in the surrounding rock to the center *O* is *r*, the hydrostatic pressure at the infinite distance of the roadway is *P*. The stress, strain and displacement of surrounding rock are analyzed according to the axisymmetric plane strain problem under the combined action of the original rock stress *P* and the support resistance $P_{s}$.

If the far-field stress disturbance effect is considered, the critical plastic depth *ρ*_*cr*_ and the critical load $P_{cr}$ can be obtained from the criterion of disturbance response of rock burst^31,32^.

1 $$ \frac{{\rho_{cr} }}{a} = \sqrt {1 + \frac{E}{\lambda } + \frac{E}{\lambda }\left( {m - 1} \right)\frac{{P_{s} }}{{\sigma_{c} }}} $$

2 $$ \frac{{P_{cr} }}{{\sigma_{c} }} = \frac{1}{m - 1}\left\{ {\frac{\lambda }{E}\left[ {1 + \frac{E}{\lambda } + \frac{E}{\lambda }(m - 1)\frac{{P_{s} }}{{\sigma_{c} }}} \right]^{{\frac{m + 1}{2}}} - \frac{\lambda }{E} - 1} \right\} $$

where *E* is the elastic modulus; *σ*_*c*_ is uniaxial compressive strength; $m = \frac{1 + \sin \varphi }{{1 - \sin \varphi }}$, *φ* is the internal friction angle;* λ* is the softening modulus.

When the support resistance is not considered, Eqs. (1) and (2) can be simplified as

3 $$ \frac{{\rho_{cr} }}{a} = \sqrt {1 + \frac{E}{\lambda }} $$

4 $$ \frac{{P_{cr} }}{{\sigma_{c} }} = \frac{{\frac{\lambda }{E} + 1}}{m - 1}\left[ {\left( {1 + \frac{E}{\lambda }} \right)^{{\frac{m - 1}{2}}} - 1} \right] $$

It can be seen from Eq. (4) that uniaxial compressive strength, elastic modulus and softening modulus are the key mechanical parameters that affect the critical load when rock burst occurs.

### The influence of uniaxial compressive strength on rock burst

Assuming that there is no support, it can be inferred from Eq. (4) that the critical load corresponding to the rock burst occurrence in the roadway is an inherent constant that depends on the properties of rock mass itself. The smaller the uniaxial compressive strength of rock mass is, the smaller the critical load is. Then, the more easily the actual load borne by rock mass reaches the critical load, the greater the possibility of rock burst is. On the contrary, the larger the critical load of rock mass is, the more difficult it is for the actual load borne by rock mass to reach the critical load, so it is less likely to experience rock burst.

### The influence of deformation modulus ratio on rock burst

The strain softening property of rock mass is the fundamental internal factor affecting rock burst occurrence^7,33^. It can be seen from Eq. (4) that the ratio of softening modulus to elastic modulus is an important factor that affects rock burst occurrence. Based on this, a new parameter for evaluating rock burst tendency-deformation modulus ratio $K_{\lambda }$-is proposed.

**Figure 2:**
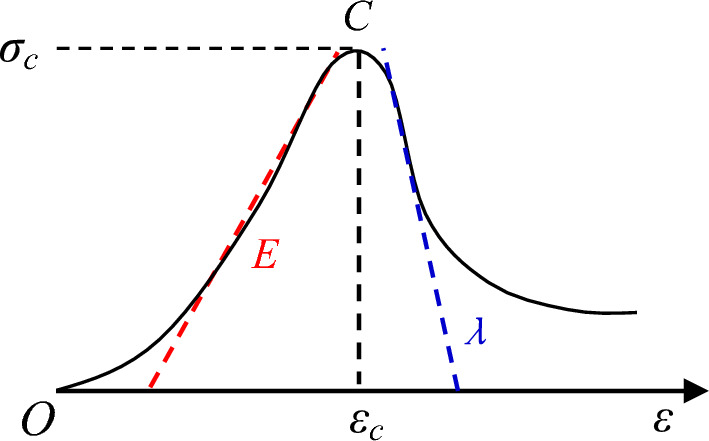
Schematic diagram of stress–strain curve of rock mass.

The stress–strain curve of rock mass can be measured by laboratory experiment, and the stress value at the peak point *C* is defined as the uniaxial compressive strength *σ*_*c*_ (see Fig. 2). The gradient of the straight line in the elastic stage before the peak point *C* is defined as the elastic modulus *E*. The gradient of the straight line in the softening stage after the peak point *C* is negative, and its absolute value is defined as the softening modulus *λ*. The softening modulus and elastic modulus are measured, and the deformation modulus ratio $K_{\lambda }$ can be expressed as:

5 $$ K_{\lambda } { = }\frac{\lambda }{E} \, $$

## Study on the size effect of uniaxial compressive strength and deformation modulus ratio

### Laboratory experimental system

**Figure 3:**
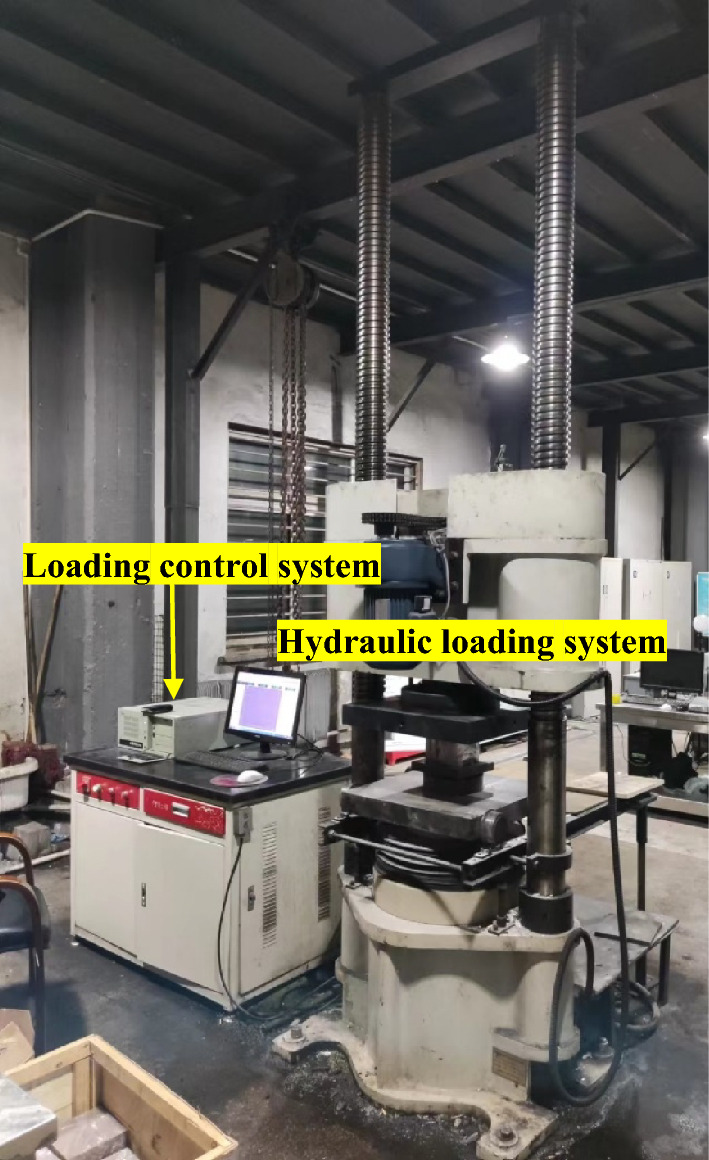
Fully automatic servo press system.

A self-developed automatic servo press system was used to study the size effect of uniaxial compressive strength and deformation modulus ratio, which mainly consists of a hydraulic loading system and a loading control system, as shown in Fig. 3. This experimental system can apply a maximum axial load of 2000 kN with an axial displacement range of 350 mm in two loading modes: displacement control and load control. The loading rate used in this experiment is 0.24 mm/min.

### Rock sample preparation

**Figure 4:**
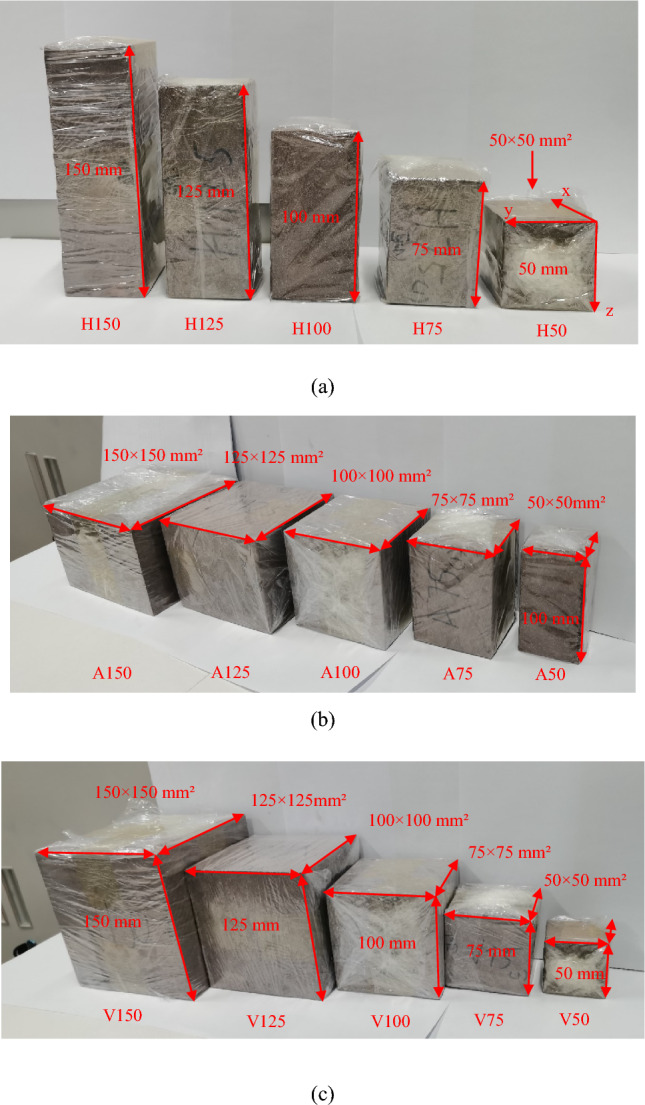
Physical images of three kinds of rock samples with different heights, cross-sectional areas and volumes: (**a**) rock samples with different heights, (**b**) rock samples with different cross-sectional areas, (**c**) rock samples with different volumes.

We conducted a series of experiments using hard brittle red sandstone. The red sandstone is cut using machine into three different types of rock samples with varying heights, cross-sectional areas and volumes, as shown in Fig. 4. For the rock samples with different heights (Fig. 4a), the cross-sectional area remains unchanged, while the height changes in increments of 25 mm. Each rock sample has a length and width of 50 mm, which is the same size as the standard rock sample. For the rock samples with different cross-sectional areas (Fig. 4b), the height of the rock samples is constant. Cross-sectional area is adjusted using length increments of 25 mm. For the rock samples with different volumes (Fig. 4c), a cubic shape is maintained and rock sample volume is adjusted using length increments of 25 mm.

The specific sizes and numbers of the three types of rock samples are shown in Table 1. The blade of the cutting machine is continuously cooled by water in the process of processing rock samples, and finally these rock samples are carefully smoothed with a grinding machine. All the polished rock samples have no visible cracks to the naked eye. The average density of the rock sample is 2.425 g/cm^3^. All rock samples are wrapped with plastic film to prevent rock fragments from popping out due to sudden destruction during the experiment, in order to ensure integrity of the rock samples after destruction, and to ensure the experiment is conducted safety.

**Table 1:** Rock sample sizes and numbers with different heights, cross-sectional areas and volumes, rock samples with equal dimensions are: H50 and V50; H100 and A50; A100 and V100.

Rock sample type	Rock sample number	Rock sample size (mm)		
		Length	Width	Height
Different heights	H50	50	50	50
	H75	50	50	75
	H100	50	50	100
	H125	50	50	125
	H150	50	50	150
Different cross-sectional areas	A50	50	50	100
	A75	75	75	100
	A100	100	100	100
	A125	125	125	100
	A150	150	150	100
Different volumes	V50	50	50	50
	V75	75	75	75
	V100	100	100	100
	V125	125	125	125
	V150	150	150	150

Red sandstone primarily comprises quartz, feldspar, calcite, and clay minerals like montmorillonite, illite, kaolinite, and chlorite. It typically exhibits two textures: granular clastic and argillaceous cement. These textures significantly influence the rock's physical and mechanical properties.

Experimental analysis reveals that the red sandstone under study exhibits a granular detrital texture. This material contains numerous large quartz particles, visible to the naked eye, ranging from 0.25 to 1.25 mm in diameter. These particles are predominantly angular, contributing to the rock’s overall strength but also imparting brittleness.

### Analysis of stress–strain curve characteristics

**Figure 5:**
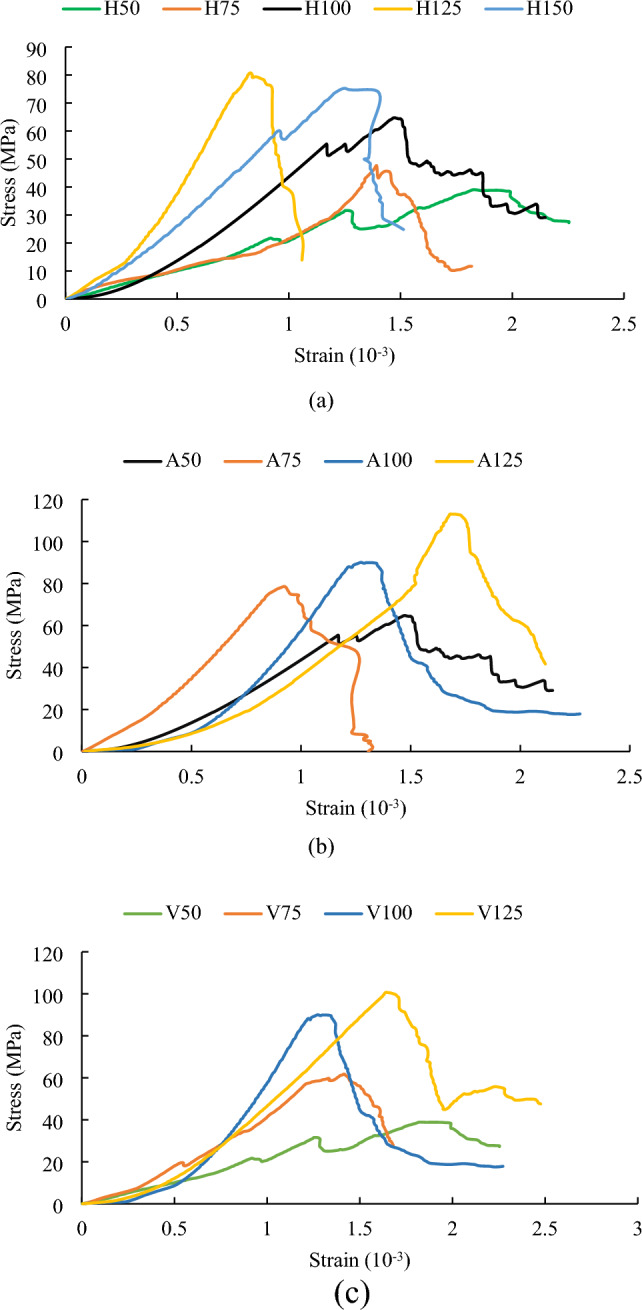
Stress–strain curves of rock samples with (**a**) different heights, (**b**) different cross-sectional areas and (**c**) different volumes.

The stress–strain curves of rock samples with different heights, cross-sectional areas and volumes are shown in Fig. 5. The complete stress–strain curves of rock samples A150 and V150 were not obtained because the maximum pressure they could withstand exceeded the maximum axial pressure that could be applied by the experimental machine. The stress–strain curve of each rock sample has roughly experienced four stages: compaction, linear elasticity, plastic deformation and failure. However, the compaction stage of all rock samples is not obvious because of the large hardness of red sandstone. When the stress does not exceed the uniaxial compressive strength, the curve presents a convex shape and the rock sample undergoes plastic deformation with the increase of strain. When the stress exceeds the uniaxial compressive strength, there is no vertical decrease in the curve and the stress does not experience a “cliff-like” drop. However, the stress decreases in a “fluctuating”' manner with the increase of strain.

The curve reflects that red sandstone has a high brittleness. The brittleness index of red sandstone calculated from the data in Fig. 5 ranges from 20.42 to 96.87, with an average value of 55.23. The brittleness index is the ratio of peak stress to peak strain. The higher the ratio, the smaller the deformation of the rock before reaching the peak stress, and the greater the brittleness.

### Size effect analysis of uniaxial compressive strength

The compressive strength of red sandstone, a fundamental mechanical property, determines its resistance to external pressure-induced damage. During a uniaxial compression test, once the applied stress surpasses this threshold, the rock will crack or fracture.

#### Analysis of uniaxial compressive strength experimental results

**Figure 6:**
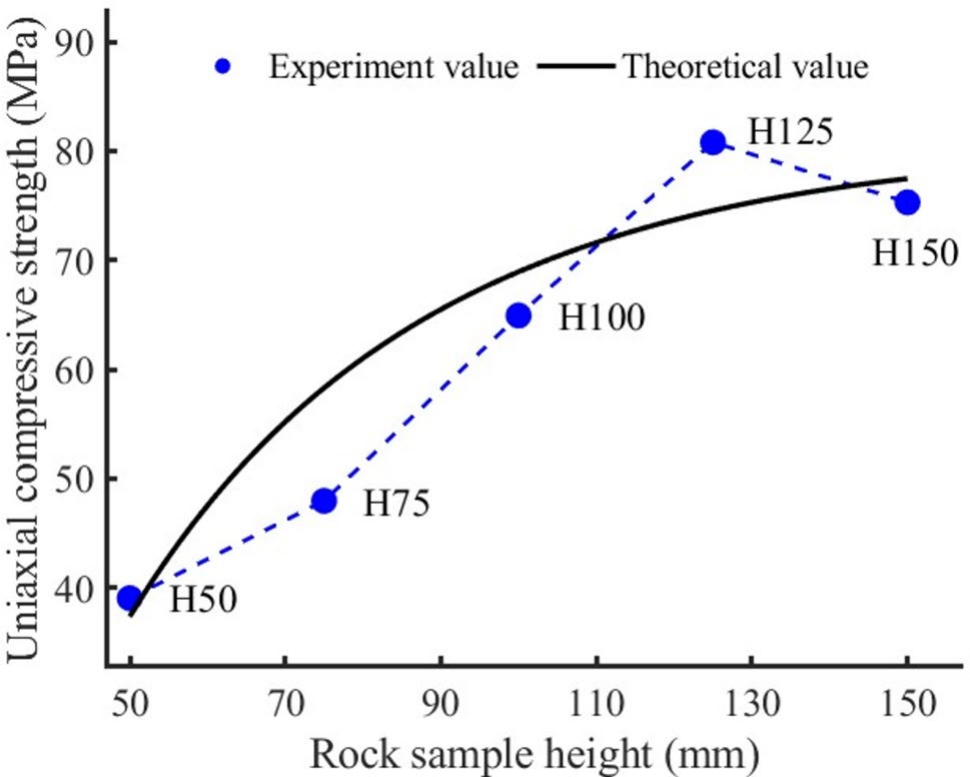
The variation trend of uniaxial compressive strength of rock samples with different heights and the comparison between theoretical value and experimental value.

It can be seen from Fig. 6 that for rock samples of different heights, the uniaxial compressive strength of rock sample H50 is the lowest, and its value is 39.04 MPa (see Table 2). The uniaxial compressive strength increases continuously with the increase of rock sample height. When the rock sample height is 125 mm, the uniaxial compressive strength reaches a maximum of 80.78 MPa. The rock sample height is increased from 50 to 125 mm, and the uniaxial compressive strength is increased by 2.07 times. Further analysis shows that for every 50 mm increase in the rock sample height, the uniaxial compressive strength increases by an average of 1.28 times. When the rock sample height increases to 150 mm, the rock sample height is three times the length of the bottom side (50 mm). At this time, the rock sample is not only subjected to compression-shear failure along the height direction after compression, but also accompanied by instability failure. Therefore, the uniaxial compressive strength decreases, and its value is 75.28 MPa.

**Table 2:** Uniaxial compressive strength, elastic modulus, softening modulus and deformation modulus ratio of rock samples with different sizes.

Rock sample type	Rock sample number	Uniaxial compressive strength (MPa)	Elastic modulus (GPa)	Softening modulus (GPa)	Deformation modulus ratio
Different heights	H50	39.04	4.23	4.27	1.01
	H75	47.95	5.42	10.91	2.01
	H100	64.92	6.03	13.31	2.21
	H125	80.78	13.61	30.02	2.2
	H150	75.28	7.08	22.12	3.12
Different cross-sectional areas	A50	64.92	6.03	13.31	2.21
	A75	78.61	11.43	20.85	1.82
	A100	90.36	13.39	23.69	1.77
	A125	113.51	8.29	13.99	1.69
	A150	–	–	–	–
Different volumes	V50	39.04	4.23	4.27	1.01
	V75	61.8	7.51	13.87	1.85
	V100	90.36	13.39	23.69	1.77
	V125	100.39	8.46	19.04	2.25
	V150	–	–	–	–

As the size of the rock sample increases, the statistical distribution probability of the micro-cracks contained in the rock sample increases, and its uniaxial compressive strength decreases. However, the results are often not absolute because of the influence of end face effect and shape effect. Based on this, the empirical formula of the relationship between uniaxial compressive strength and the ratio of height to width proposed by Zhu et al.^34^

6 $$ \sigma_{c} = \sigma_{02} \,  exp[ - (2 - H/D)a] $$

where $\sigma_{c}$ is the uniaxial compressive strength; $\sigma_{02}$ is the uniaxial compressive strength of rock sample with height to width ratio of 2; *a* is the undetermined coefficient, whose value is related to lithology, experimental conditions, etc. According to reference^35^, *a* is 0.791.

**Figure 7:**
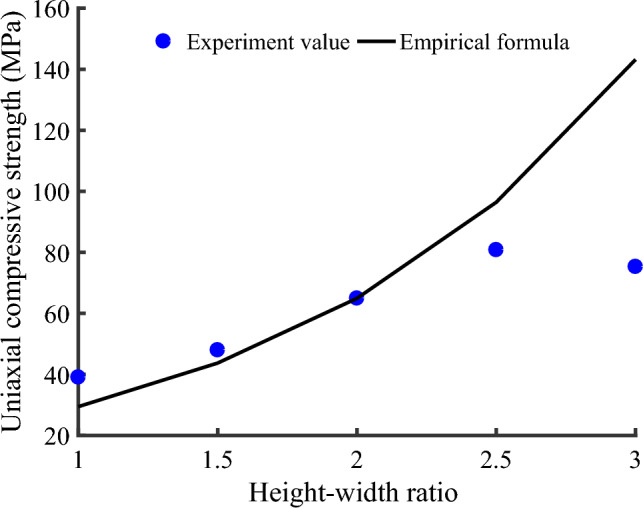
Comparison of experimental values and empirical values of uniaxial compressive strength of rock samples with different heights.

Figure 7 is the comparison between the experimental value of uniaxial compressive strength of rock samples with different heights and the theoretical value of Eq. (6). When the height of the rock sample does not exceed 125 mm, that is, the ratio of height to width does not exceed 2.5, the experimental value of uniaxial compressive strength closely matches the theoretical value, which indicates the correctness and reliability of the experimental results. As the ratio of height to width approaches 3, the experimental value becomes quite different from the theoretical value, and the Eq. (6) is no longer applicable.

**Figure 8:**
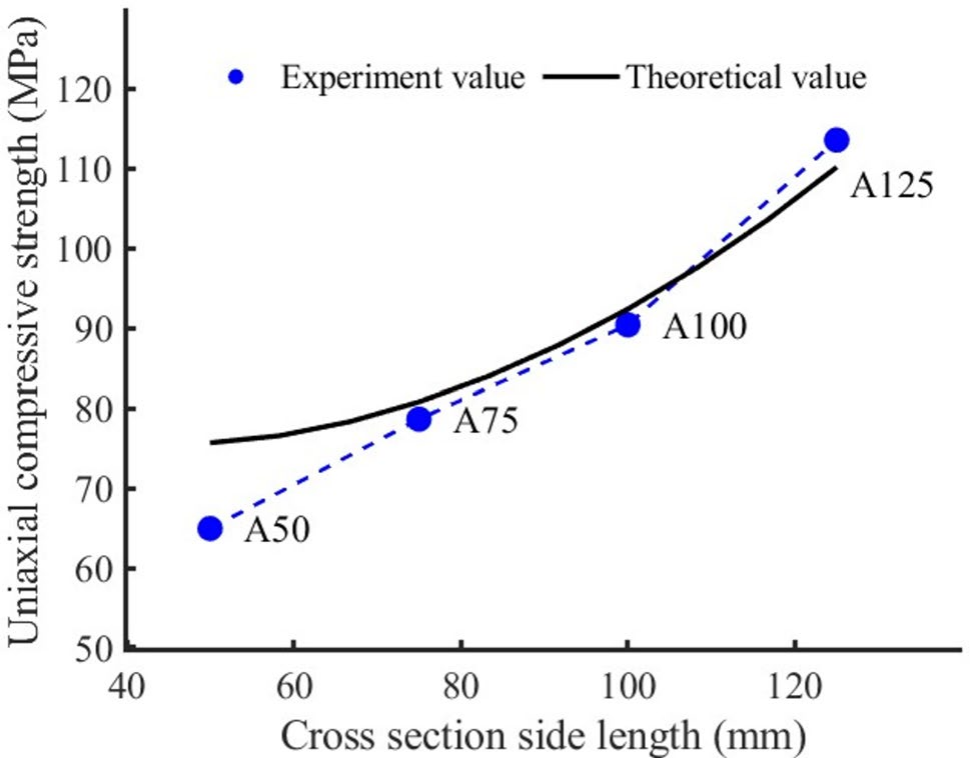
The variation trend of uniaxial compressive strength of rock samples with different cross-sectional areas and the comparison between theoretical value and experimental value.

It can be seen from Fig. 8 that for rock samples with different cross-sectional areas, the uniaxial compressive strength of rock sample A50 is the lowest, whose value is 64.92 MPa (see Table 2), the uniaxial compressive strength increases continuously with increasing cross-sectional area of rock sample. When the cross-sectional side length reaches 125 mm, the uniaxial compressive strength reaches a maximum of 113.51 MPa. The change represents an increase in uniaxial compressive strength by a factor of 1.77. Through further analysis, it can be seen that the uniaxial compressive strength increases by an average of 1.21 times for every 50 mm increase in the cross-sectional side length.

**Figure 9:**
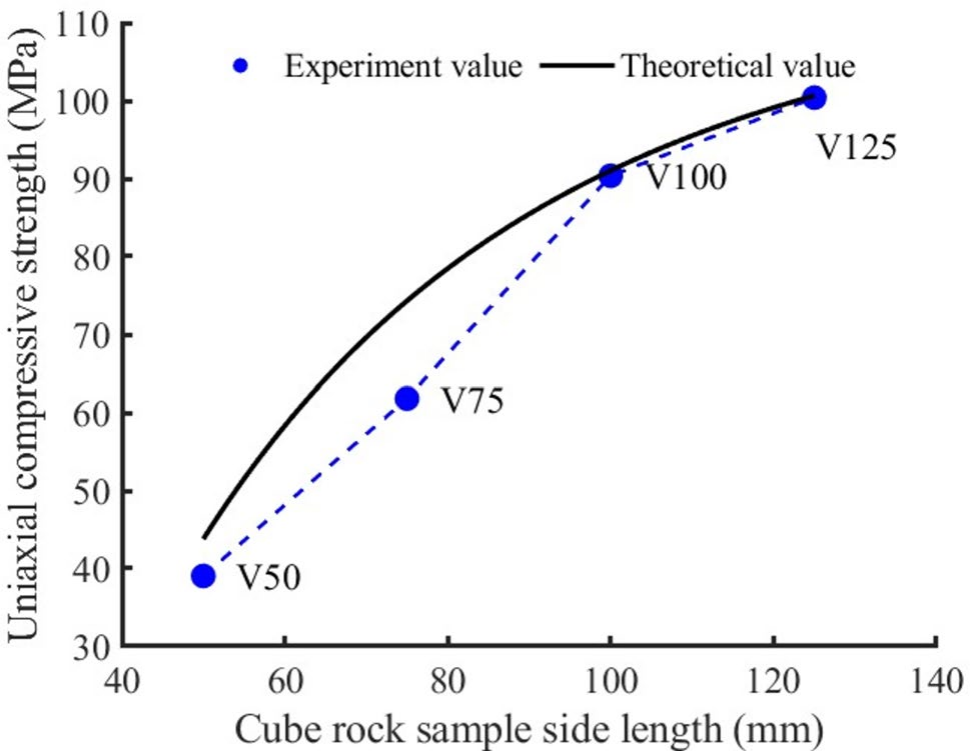
The variation trend of uniaxial compressive strength of rock samples with different volumes and the comparison between theoretical value and experimental value.

It can be seen from Fig. 9 that the uniaxial compressive strength of rock sample V50 is the lowest for cuboid rock samples of different volumes, which is 39.04 MPa (see Table 2). The uniaxial compressive strength increases continuously with the increase of rock sample volume. When the side length reaches 125 mm, the uniaxial compressive strength reaches a maximum of 100.39 MPa. The change in side length represents an increase in uniaxial compressive strength by a factor of 2.57. In addition, the uniaxial compressive strength of rock sample V75 is 1.58 times that of rock sample V50, the uniaxial compressive strength of rock sample V100 is 1.46 times that of rock sample V75, and the uniaxial compressive strength of rock sample V125 is 1.11 times that of rock sample V100. Therefore, the increase of uniaxial compressive strength decreases with the increase of rock sample volume. Through further analysis, it can be seen that the uniaxial compressive strength increases by an average of 1.39 times for every 50 mm increase in the side length of rock sample.

#### Theoretical analysis of size effect of uniaxial compressive strength

According to reference^34^:

7 $$ \sigma = E\varepsilon e^{{( - \varepsilon /\varepsilon_{0} )}} $$

The linear strain is

8 $$ \varepsilon = \frac{{{\Delta }l}}{{l_{0} }} $$

where *l*_0_ is the original height of rock sample, $\Delta l$ is deformation amount.

${\Delta }l$ consists of two parts:

9 $$ \Delta l = \Delta l_{1} + \Delta l_{2} $$

where $\Delta l_{1}$ is elastic deformation, $\Delta l_{2}$ is plastic deformation.

$\Delta l_{1}$ and $\Delta l_{2}$ can be written as:

10 $$ \Delta l_{1} = (\sigma l_{0} )/E $$

11 $$ \Delta l_{2} = \frac{\sigma }{Y} $$

where *Y* is weakening coefficient, $Y = \frac{d\sigma }{{d{\Delta }l_{2} }}$, it represents the strength reduction of rock samples caused by unit plastic deformation. It can be seen from the reference^35^ that the weakening coefficient *Y* is 500 MPa/mm.

Bring Eqs. (10) and (11) into Eq. (9) at the same time, we have

12 $$ \varepsilon = \frac{\sigma }{E} + \frac{\sigma }{YDx} $$

Bring Eq. (12) into Eq. (7), we have

13 $$ \sigma = - \frac{{\varepsilon_{0} EYDx}}{E + YDx}\ln \left( {\frac{YDx}{{E + YDx}}} \right) $$

where *D* is the cross-sectional side length, *x* is the ratio of height to width. *X* is the variable in the case of rock samples with different heights as *D* remains constant. *D* is the variable because *x* of all rock samples is 1 for rock samples with different volumes. Both *x* and *D* are variables for rock samples with different cross-sectional areas.

It can be seen from Eq. (13) that the uniaxial compressive strength is a function of elastic modulus, side length of the compression surface and the ratio of height to width. The theoretical and experimental values of uniaxial compressive strength of rock samples with different heights, different cross-sectional areas and different volumes are compared in Figs. 6, 8, 9. The comparison results show that the proposed theoretical values are close to the experimental values, that is, the theoretical analysis curve fits the change trend of uniaxial compressive strength well.

In order to evaluate the quality of the model, we calculated the mean square error (MSE) and the root mean square error (RMSE). The smaller their values, the stronger the interpretation ability of independent variables (elastic modulus, compression surface side length, height) to the dependent variable (uniaxial compressive strength), and the higher the prediction accuracy of the model. For sample H (MSE): 33.946965, (RMSE): 5.826402; For sample A (MSE): 33.818994, (RMSE): 5.815410; For V sample (MSE): 44.878400. (RMSE): 6.699134. It can be seen that the model can predict the uniaxial compressive strength well.

### Analysis of size effect of deformation modulus ratio

**Figure 10:**
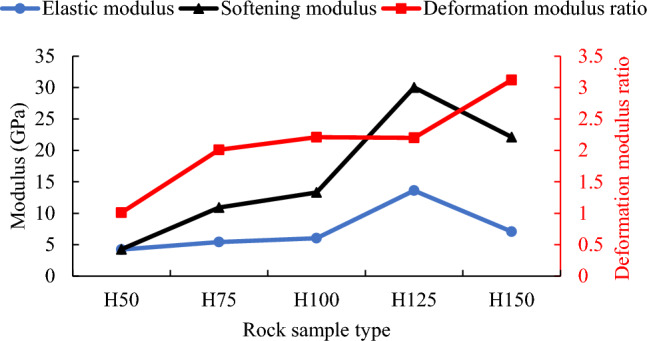
The change curves of elastic modulus, softening modulus and deformation modulus ratio of rock samples with different heights.

When the rock sample height is between 50 and 100 mm, the elastic modulus and softening modulus increase slowly with the increase of the rock sample height, as shown in Fig. 10. When the height increases to 125 mm, the elastic modulus and softening modulus both significantly increase and reach their maximum values of 13.61 MPa and 30.02 MPa respectively (see Table 2). When the height increases from 100 to 125 mm, both the elastic modulus and softening modulus increase by 1.26 times. However, the elastic modulus and softening modulus are greatly reduced when the height is 150 mm, and the values are reduced to 7.08 MPa and 22.12 MPa respectively. The elastic modulus and softening modulus of rock sample H150 are about 1/2 and 2/3 of that of rock sample H125. Through further analysis, it can be seen that the softening modulus of each rock sample is greater than the elastic modulus.

It can be seen from Fig. 10 that the deformation modulus ratio of rock sample H50 is the lowest, whose value is 1.01. The deformation modulus ratio increases with the increase of the height. The deformation modulus ratio of rock sample H150 is the largest, at 3.12. Therefore, the deformation modulus ratio also increases by 2 times when the height increases by 2 times. When the height changes in the range of 75–125 mm, the deformation modulus ratio does not change significantly, and its average value is 2.14.

**Figure 11:**
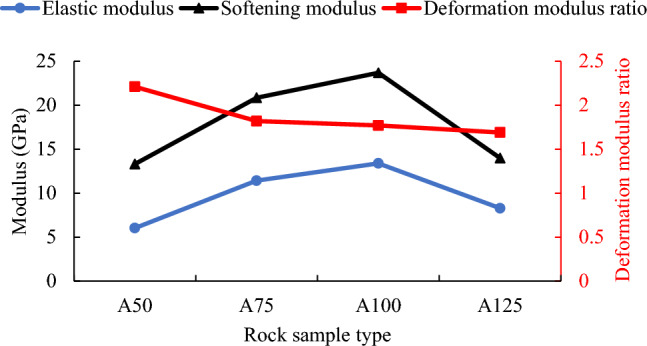
The change curves of elastic modulus, softening modulus and deformation modulus ratio of rock samples with different cross-sectional areas.

It can be seen from Fig. 11 that when the cross-sectional side length of the rock sample increases from 50 to 100 mm, the elastic modulus increases from 6.03 to 13.39 MPa and the softening modulus increases from 13.31 to 23.69 MPa. The elastic modulus and softening modulus increased by factors of 1.22 and 0.77 respectively. When the cross-sectional side length increases from 100 to 125 mm, the elastic modulus and softening modulus decrease to 8.29 MPa and 13.99 MPa respectively. Further analysis shows that the softening modulus of each rock sample is greater than the elastic modulus. Both elastic modulus and softening modulus of rock sample A125 are about 3/5 of that of rock sample A100.

The deformation modulus ratio of rock sample A50 is the largest, with a value of 2.21. As the cross-sectional area increases, the deformation modulus ratio continues to decrease. When the cross-sectional side length increases from 75 to 125 mm, the deformation modulus ratio decreases from 1.82 to 1.69, only decreasing by 0.13. It can be seen that when the cross-sectional side length changes in the range of 75–125 mm, the deformation modulus ratio can be approximately considered to remain unchanged, and its average value is 1.76.

**Figure 12:**
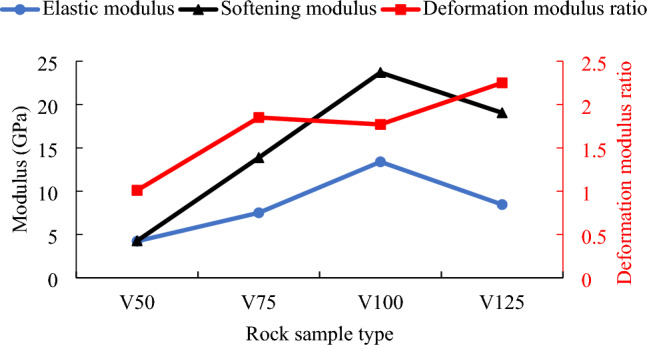
The change curves of elastic modulus, softening modulus and deformation modulus ratio of rock samples with different cube volumes.

It can be seen from Fig. 12 that when the side length of the cubic rock sample type increases from 50 to 100 mm, both elastic modulus and softening modulus increase linearly. Because the linear slope of softening modulus is greater than that of elastic modulus, the increase rate of softening modulus is greater than that of elastic modulus. Moreover, the elastic modulus increases from 4.23 to 13.39 MPa and the softening modulus increases from 4.27 to 23.69 MPa, respectively. The elastic modulus and softening modulus increase by 2.17 and 4.56 times respectively. When the side length of the cube rock sample increases from 100 to 125 mm, the elastic modulus and softening modulus decrease to 8.46 MPa and 19.04 MPa respectively, with a decrease of 4.93 MPa and 4.65 MPa respectively. The elastic modulus and softening modulus of rock sample V125 are about 3/5 and 4/5 of that of rock sample V100 respectively. Further analysis shows that the softening modulus of each rock sample is greater than the elastic modulus. The deformation modulus ratio of different volume rock samples shows an upward trend in general. The deformation modulus ratio of rock sample V50 is the smallest, which is 1.01. The deformation modulus ratio of rock sample V125 is the largest, which is 2.25.

## The influence of rock size on the energy conversion law during rock failure process

**Figure 13:**
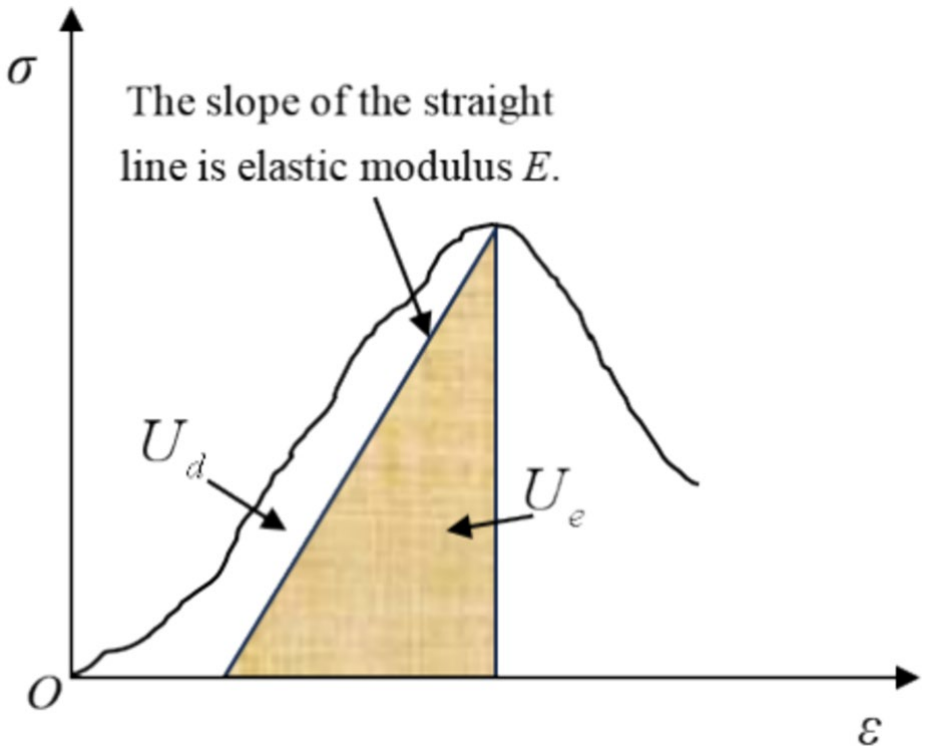
Quantitative relationship of energy release and energy dissipation.

The complexity of the internal components of rock mass determines the diversity of energy conversion during the failure process. According to the proportion of various energy before rock failure, energy can be divided into elastic energy and dissipated energy^36–40^, as shown in Fig. 13.

The rock undergoes deformation through external forces. It is assumed that there is no heat exchange between the rock and the external environment during the deformation process, and the total input energy generated by the external work is *U*. According to the first law of thermodynamics, we have

14 $$ U = U_{d} + U_{e} = \int_{0}^{{\varepsilon_{c} }} {\sigma d\varepsilon } = \int_{0}^{{\varepsilon_{c} }} {E\varepsilon e}^{{ - \frac{\varepsilon }{{\varepsilon_{0} }}}} d\varepsilon $$

15 $$ U_{e} = \frac{1}{2}\sigma_{c} \varepsilon_{e} = \frac{{\sigma_{c}^{2} }}{2E} $$

where *U* is the total energy density, $U_{d}$ is the dissipated energy density, $U_{e}$ is the elastic energy density, $\varepsilon_{e}$ is the elastic strain. Dissipated energy is used to form internal damage and plastic deformation of rock mass, and its change satisfies the second law of thermodynamics, that is, the change of internal state conforms to the trend of entropy increase.

By integrating Eq. (7), the total energy *U* absorbed by rock mass before the peak can be expressed as follows:

16 $$ U = \mathop \smallint \nolimits_{0}^{{\varepsilon_{1} }} \sigma d\varepsilon = \mathop \smallint \nolimits_{0}^{{\varepsilon_{1} }} E\varepsilon e^{{ - \frac{\varepsilon }{{\varepsilon_{0} }}}} d\varepsilon = ( - E\varepsilon_{0} \varepsilon_{1} - E\varepsilon_{0}^{2} )e^{{ - \frac{{\varepsilon_{1} }}{{\varepsilon_{0} }}}} + E\varepsilon_{0}^{2} $$

The elastic energy density is calculated as follows:

17 $$ U_{e} = \frac{1}{2}\sigma_{c} \varepsilon_{e} = \frac{1}{2}E\varepsilon_{c}^{2} e^{{ - \frac{{2\varepsilon_{c} }}{{\varepsilon_{0} }}}} $$

The dissipated energy density is calculated as follows:

18 $$ \begin{aligned} U_{d} & = U - U_{e} = \left( { - E\varepsilon_{0} \varepsilon_{c} - E\varepsilon_{0}^{2} } \right)e^{{ - \frac{{\varepsilon_{c} }}{{\varepsilon_{0} }}}} - \frac{1}{2}E\varepsilon_{c}^{2} e^{{ - \frac{{2\varepsilon_{c} }}{{\varepsilon_{0} }}}} + E\varepsilon_{0}^{2} \\ & = \left[ { - E\varepsilon_{0} \left( {\frac{{\sigma_{c} }}{E} + \frac{{\sigma_{c} }}{YDx}} \right) - E\varepsilon_{0}^{2} } \right]e^{{ - \left( {\frac{{\sigma_{c} }}{{E\varepsilon_{0} }} + \frac{{\sigma_{c} }}{{YDx\varepsilon_{0} }}} \right)}} - \frac{1}{2}E\left( {\frac{{\sigma_{c} }}{E} + \frac{{\sigma_{c} }}{YDx}} \right)^{2} e^{{ - 2\left( {\frac{{\sigma_{c} }}{{E\varepsilon_{0} }} + \frac{{\sigma_{c} }}{{YDx\varepsilon_{0} }}} \right)}} + E\varepsilon_{0}^{2} \\ \end{aligned} $$

It can be seen from Eq. (18) that the dissipated energy *U*_*d*_ is a function of the elastic modulus *E*, the material parameter *ε*_*0*_, the uniaxial compressive strength *σ*_*c*_ and the height to width ratio *x*. Since we derive the relationship between compressive strength and size in section “Theoretical analysis of size effect of uniaxial compressive strength”, we actually obtain the functional relationship between rock energy change and rock size.

The elastic energy ratio refers to the percentage of the ratio of elastic energy density to total energy density, and the dissipated energy ratio refers to the percentage of the ratio of dissipated energy density to total energy density.

**Figure 14:**
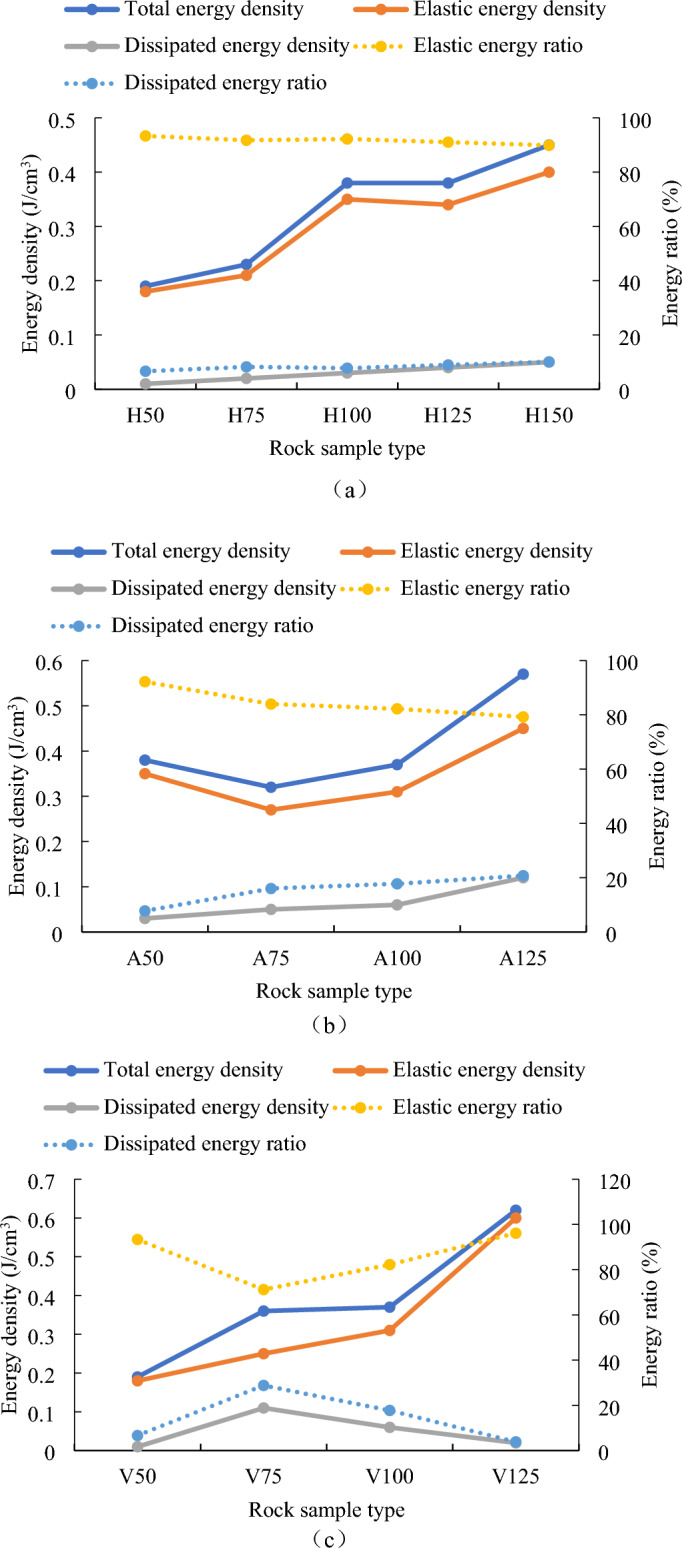
Various energy trends of rock samples with (**a**) different heights, (**b**) different cross-sectional areas and (**c**) different cube volumes.

As shown in Fig. 14a, the total energy density and elastic energy density of rock samples with different heights both increase in a stepped way. The total energy density and elastic energy density of rock sample H50 are the smallest, at 0.19 J/cm^3^ and 0.18 J/cm^3^ respectively (see Table 3). When the sample height is increased to 100mm, the total energy density and elastic energy density increase to 0.38 J/cm^3^ and 0.35 J/cm^3^ respectively. The total energy density and elastic energy density of rock sample H150 are 2 times and 1.94 times that of rock sample H50 respectively. When the rock sample height increases by 125 mm, the two energy densities decrease slightly. When the rock sample height continues to increase, the total energy density and elastic energy density reach the maximum of 0.45 J/cm^3^ and 0.4 J/cm^3^ respectively. The dissipated energy density and the proportion of dissipated energy both linearly increase with the increase in rock sample height, while the elastic energy density shows a linear decrease with the increase in rock sample height.

**Table 3:** Various energies of rock samples with different heights, different cross-sectional areas and different volumes.

Rock sample number	Total energy density J/cm^3^	Elastic energy density J/cm^3^	Dissipated energy density J/cm^3^	Elastic energy ratio %	Dissipated energy ratio %
H50	0.19	0.18	0.01	93.35	6.65
H75	0.23	0.21	0.02	91.75	8.25
H100	0.38	0.35	0.03	92.22	7.78
H125	0.38	0.34	0.04	91.05	8.95
H150	0.45	0.4	0.05	89.90	10.10
A50	0.38	0.35	0.03	92.22	7.78
A75	0.32	0.27	0.05	83.98	16.02
A100	0.37	0.31	0.06	82.24	17.76
A125	0.57	0.45	0.12	79.25	20.75
V50	0.19	0.18	0.01	93.35	6.65
V75	0.36	0.25	0.11	71.19	28.81
V100	0.37	0.31	0.06	82.24	17.76
V125	0.62	0.6	0.02	96.12	3.88

As shown in Fig. 14b, when the cross-sectional side length of rock sample increases from 50 to 75 mm, both the total energy density and the elastic energy density decrease. Then, the two energy densities increase with the increase of the cross-sectional side length. The total energy density and elastic energy density of rock sample A75 are the smallest, at 0.32 J/cm^3^ and 0.27 J/cm^3^ respectively (see Table 3). The total energy density and elastic energy density of rock sample A125 are the largest, which are 0.57 J/cm^3^ and 0.45 J/cm^3^ respectively. The dissipated energy density and the dissipated energy ratio increase with the increase of cross-sectional side length, while the elastic energy density decreases with the increase of cross-sectional side length.

As shown in Fig. 14c, the total energy density and elastic energy density of cubic rock samples with different volumes generally increase with the increase of rock sample volume. When the side length increases from 100 to 125 mm, the total energy density and elastic energy density increase greatly. The total energy density has increased from 0.37 to 0.62 J/cm^3^ (see Table 3), and the elastic energy density has increased from 0.31 to 0.6 J/cm^3^.

When the side length increases from 50 to 75 mm, both the dissipated energy density and the dissipated energy ratio increase. Then, the two energy densities decrease linearly with the increase of the side length of cubic rock sample. The dissipated energy density and dissipated energy ratio of rock sample V75 are the largest, with values of 0.11 J/cm^3^ and 28.81%, respectively. The rock sample V50 has the lowest dissipated energy density, which is 0.01 J/cm^3^. The dissipated energy ratio of rock sample V125 is the smallest, with a value of 3.88%. Both the elastic energy ratio and the dissipated energy ratio show the opposite deformation law, which first decrease and then increase. The elastic energy ratio of rock sample V75 is the smallest, with a value of 71.19%. The elastic energy ratio of rock sample V125 is the highest, with a value of 96.12%.

In red sandstone samples of different sizes, discontinuity and fracture have different influence on mechanical parameters, which leads to different rock burst tendency.

In smaller samples, the presence of cracks or discontinuities has a greater impact on the strength and disintegration properties of the rock, because the presence of cracks has a greater impact on the whole. When the number and scale of discontinuities and fractures are relatively small, the rock burst tendency is relatively low because of their small influence on stress concentration. When the number and scale of discontinuities and cracks are relatively large, the rock burst tendency is relatively high, but due to the small energy storage capacity, the rock burst level is low.

In larger size samples, the overall strength and stability of the rock may be more dependent on its overall structure and other factors. However, discontinuities and fractures may be more complex and diversified, and these structural features have more significant effects on stress concentration, thus increasing the rock burst tendency and rockburst intensity.

## Failure mode analysis

A part of the energy absorbed by the rock is dissipated due to the sliding friction of the fracture surface in the process of uniaxial compression, while the remaining part is converted into elastic energy and stored in the rock. The elastic energy is released instantaneously in the post-peak failure stage. It can be seen from the above section that the energy storage limit of large-size rock samples with wide and flat shape (such as A125, V125) is smaller than that of thin and long rock samples (such as H75, H100).

**Figure 15:**
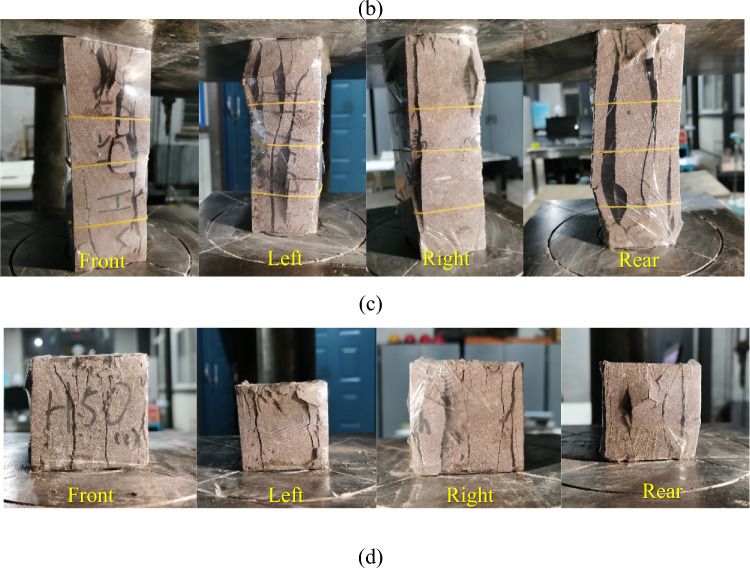
Fracture morphology of rock samples under uniaxial compression: (**a**) Rock sample A125, (**b**) Rock sample H100/A50, (**c**) Rock sample H50, (**d**) Rock sample H50/A50.

Therefore, the large-sized rock samples with a wide and flat shape release a larger amount of energy at the moment of failure, and the degree of damage is more severe, as shown in Fig. 15a.

**Figure 16:**
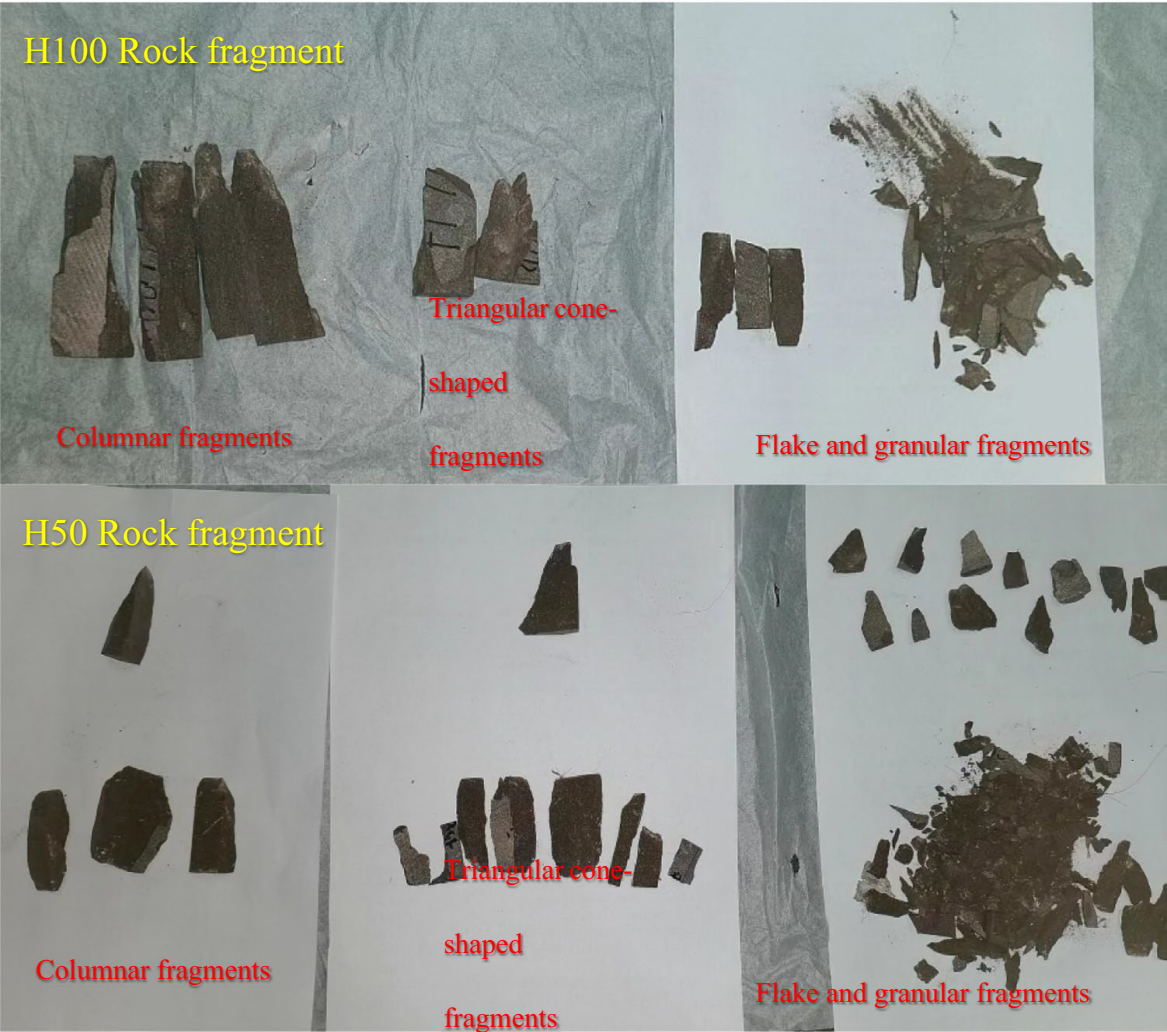
Distribution of rock sample fragments.

The energy absorption tends to be gentle when the rock sample height is 100 mm for the rock samples with variable pressure height. The rupture morphology of this rock sample is mainly splitting, there is a certain angle between the direction of crack propagation and the direction of the principal stress and a shear failure surface at the edge of the end face. After the failure of the rock sample, the central fragments are mostly cylindrical or sheet-shaped, and the fracture degree of rock sample is small, as shown in Figs. 15b and 16. The rock samples with large height and width ratio (such as rock samples H125 and H150) are mainly broken in the form of splitting, the rock sheet similar to “pressure rod instability” appears on the side of the rock sample, as shown in Fig. 15c, and the failure pattern of the rest of the rock sample is the same as that of rock sample H100.

Due to the influence of end face effect, the rupture morphology of the smaller rock sample is more complex^41,42^. The edges and corners of the fractured rock sample are mostly triangular conical fragments, and there are multiple shear failure planes throughout the rock sample in the middle of rock sample. For example, the fragments are mostly cylindrical or sheet-shaped after the destruction of rock sample H50, and there are obvious shear planes at the corners and middle parts, as shown in Figs. 15d and 16.

For rock samples with compression area as a variable, the fracture degree of rock samples decreases with the increase of size, and the broken fragments are larger. Because the dissipated energy accounts for a larger proportion and the stored elastic energy is also more, the failure is accompanied by small rock burst for larger rock samples (such as A125). In addition, there are shear cracks in the middle of the damaged rock sample, a funnel-shaped and conical rupture rock block with the end face as the bottom appears in the center of one end after the failure of rock sample, and a tensile failure along the axis is generated at the top of the cone.

The fracture form is the same as the above rock sample H50 for the rock samples with the simultaneous change of compression area and compression height, but the splitting failure in the middle of rock sample increases.

The failure mechanism of red sandstone under uniaxial compression is closely related to the sample size, which can significantly affect the rock burst tendency.

For small-size samples, as the influence of cracks and heterogeneity on the size effect is dominant, the failure is often more localized and uneven. The common failure mode is tensile shear mixed failure, which is manifested as both vertical splitting caused by tension and oblique shear failure.

For wide and flat medium-size samples, due to the large volume, the influence of cracks and heterogeneity on size effect decreases, while the influence of end effect on size effect increases. Further study found that the end effect produced a “self-confining pressure” effect on the samples, which greatly improved the compressive strength of the rocks and also increased the elastic energy of rock storage. When the samples were damaged, A large amount of the accumulated elastic energy is suddenly released in the rock, forming a small “rock burst”.

For large-size samples with increased height, the influence of the size effect decreases due to cracks and heterogeneity, and the middle of the sample is far away from the end of the sample, resulting in a gradual decrease of the influence of the end effect. At this time, the strength of the sample gradually tends to be stable, but the sample is still dominated by vertical splitting due to the brittleness of red sandstone and vertical cracking induced by the end effect. Transverse cracks can be seen in the middle of the specimen.

## Conclusion

1. The mechanical model of rock burst roadway is constructed. It is pointed out that uniaxial compressive strength and deformation modulus ratio are the two most critical mechanical parameters affecting the critical load when rock burst occurs.
2. The smaller the uniaxial compressive strength is, the smaller the critical load is. The more easily the actual load on rock mass reaches the critical load, the more easily the rock burst occurs. On the contrary, rock burst is not easy to occur. The smaller the deformation modulus ratio is, the weaker the brittleness is, and the less likely rock burst is to occur. On the contrary, rock burst is more likely to occur.
3. The functional relationship between uniaxial compressive strength and elastic modulus, side length of compression surface and height to width ratio is obtained, the correctness of this function relationship is verified by comparing the experimental values with the theoretical values. Both theoretical analysis and experimental results show that the uniaxial compressive strength of rock samples with different heights, cross-sectional areas and cubic-shaped volumes increase with the increase of rock sample size.
4. The deformation modulus ratio of rock samples with different heights and cubic-shaped volumes show an upward trend on the whole, while that of rock samples with different cross-sectional areas show a downward trend on the whole. The elastic modulus and softening modulus of the three types of rock samples show a trend of initial increasing then decrease. Among the rock samples of different heights, the elastic modulus and softening modulus of rock sample H125 are the largest, which are 13.61 MPa and 30.02 MPa respectively. Among the rock samples with different cross-sectional areas and different cubic-shaped volumes, the elastic modulus and softening modulus of rock samples A100 and V100 are the largest, and their values are 13.39 MPa and 23.69 MPa respectively. Moreover, the softening modulus of each rock sample is greater than the elastic modulus.
5. The functional relationship between rock energy and rock size is obtained. The change of compression height and compression area will affect the storage of rock energy and the release of elastic energy, and then change the degree and form of rock fracture. The fracture of rock sheet similar to “pressure rod instability” will occur at the same time of the splitting in the case of elongated rock sample, while large-size rock samples with wide and flat shape are more prone to impact damage.

## Data Availability

The data used to support the findings of this study are available from the corresponding author upon request.
